# Boston Letter

**Published:** 1881-02

**Authors:** 


### Domestic Correspondence.

---

### ARTICLE VII.

### BOSTON LETTER.

*Messrs. Editors:*

We are in the midst of a noble rain storm. The water falls with an abundance so copious as to be a cause for gladness and gratitude. I think I am not mistaken in saying that we have not enjoyed such a rain for several months. Throughout New England, water has been almost a luxury since July last. In Boston those who are at all particular as to the quality of their drinking water, buy for the table, Belmont, Apollinaris, Poland or some other spring water. The Cochituate has a vile flavor, and, while apparently clear and bright, leaves, upon a properly constructed filter, matters too numerous to mention, and upon the palate a taste which reminds one of old paint pots; sometimes of the hold of a fishing smack. What share, if any, such water may have in current illness, I am unable to say. We know that the disagreeable quality of the water is due to the fact that the lakes which supply us are very low in quantity, and, doubtless, as we approach the bottom of a lake, the less pure do we find the water. Nevertheless, I cannot learn that any class of diseases which might be increased by impure water is notably prominent. But we need rain in abundance. If it should not come before farmers plant in the spring, our crops will suffer and, it may be, when the tonic of winter weather is gone, that we shall begin to see the effects of unwholesome water upon the people. Moreover, we have had the unique experience of enjoying a moderate temperature while cities far to the south of us, Charleston, Savannah, etc., have suffered an unknown frigidity. Something ails New England weather. For the past six months it has been positively unnatural. Vennor, the new prophet, is made responsible for it all.

The abuse and over-use of dispensaries, form just now a subject which is being warmly discussed by our medical societies. Opinions present a most curious unlikeness. Some physicians favor a great stringency and the appointment of salaried inspectors whose duty shall be that of discovering how able or unable patients may be to pay for advice. Others, notably the specialists, think if anything be done to decrease the number of patients at dispensaries, whose clinical material is used for purposes of instruction, medical education will suffer. These are the two extremes. No definite step has been taken to modify a trouble which, with us at least, has assumed a very serious and abnormal shape.

A new annoyance has appeared in the form of an abortion called the College of Physicians and Surgeons of Boston. The chief demon is a vendor of masonic paraphernalia, who has voted himself a doctor, and who apparently intends to try the Buchanan curriculum. It is a legitimate outgrowth of the failure of the mismanaged medical bill of last winter, which, while as necessary as pure water, was sprung upon us in a form too imperfect for success. At the outset the impulse of every right-minded physician was to indorse it, but upon sober second thought and candid consideration, it was found that the provisions of the bill were full of errors, and it was dropped. Thereupon the quack taketh with him seven other spirits more wicked than himself, * * and the last state of that man is worse than the first. We did not even scotch the snake. Consequently, bogus medicine develops a cheap school. Boldness becomes effrontery. It would be a strange condition of things if diplomas were to be obtained in Boston for mere money. But as regards quackery, we are in that helpless state of which the less said the better.

The feeling of annoyance which naturally arises when this subject is mentioned, suggests another matter, of which, on the contrary, too much cannot be said: You will recall the series of Health Primers published by Lindsay & Blakiston, of Philadelphia. The stereotype plates of six of these were sold some months ago by Mr. Blakiston (who now holds exclusive possessorship of these books) to Ward & Lock, a firm of English publishers. In concluding the sale Mr. Blakiston especially stipulated that the names of the editor of the series, Dr. W. W. Keen, and also of the authors, should all appear in the English reprints. The result was that this high minded firm of Ward & Lock issued the books *anonymously* under the title of "Ward & Lock's Long Life Series, accurately written and carefully edited by distinguished members of the medical profession;" leaving it to be inferred that the authors were British. In reply to Mr. Blakiston's remonstrance, these noble scions of Britannia replied that they had a perfect right to the course they had taken, and moreover, that "*so many changes* were requisite to adapt the books to the English market, that it *would have been unjust to use the authors' names!*" These so-called changes were merely necessary to cover American allusions and were not only unworthy of mention, but had no bearing whatever upon the intrinsic character of the primers. This is not the first offense of this redoubtable firm. It is said that John Habberton, learning that "Helen's Babies" had a large English sale without copyright, went into Canada and there wrote the last chapter of his next book, thus securing both English and American copyright. Whereupon, Messrs. Ward & Lock had a new chapter witten in the pla[illegible]e of Habberton's last chapter, and then published the book in England in spite of the copyright. Comment upon such trickiness merely vexes one without producing the slightest alteration in the moral quality of sharpers like these Englishmen.

Dr. John Homans, on Monday evening last, read before the Boston Society for Medical Improvement, the history of twenty-five cases of ovariotomy upon which he has operated within twelve months, with the remarkable result of twenty-three recoveries and only two deaths, these undoubtedly being hastened by the extremely sultry weather of last summer, at which time they occurred. One of the cases of recovery was rendered unique by the fact that subsequent to the operation the chest was found to contain fluid. By aspiration forty ounces of flui l were removed. This in no way retarded the convalescence.

In a second case both ovaries were removed with the tumor.

In spite of this menstruation reappeared within a short time after recovery and has regularly continued. As Dr. Homans remarked, this phenomenon has a bearing on the Battey operation. These cases were all treated antiseptically, and Dr. Homans, who seems to possess the peculiar qualifications which apparently are a *sine quâ non* in successful ovariotomy, has more than reason to be proud of his remarkable and brilliant results. The immense strides which this operation has taken within a very few years, and the almost absolute certainty of its success in the hands of skilled and experienced men, is very significant. I was told last night that not longer than ten years ago, Dr. Homans himself positively refused to do ovariotomy because of his lack of faith in its safety. To-day, his success has given him a well-won fame. He is a courageous operator, very particular as to details, especially thorough in sponging and cleansing the abdominal cavity, and unusually careful in the after-treatment. He attributes his good fortune in operation to Listerism. I may add that he never uses catgut ligatures, but confines himself strictly to carbolized silk which he applies to the pedicle behind the clamp, which he uses only temporarily, removing it after the ligature is firmly in place.

You will have noticed that Dr. George B. Shattuck has been appointed editor of the *Boston Medical and Surgical Journal*, *vice* Dr. J. C. Warren, who resigned after giving the *Journal* a fresh life, unusual vigor and a handsome form and dress. Those who know Dr. Shattuck do not doubt that the development of the *Journal* will steadily continue under his influence. Dr. James C. White, to whose unusually able leadership much of the recent success of the Boston Society for Medical Improvement is due, has declined re-nomination as president, much to the hearty regret of the Society.

BOSTON, January 12, 1881.

